# Gait abnormalities and longitudinal fall risk in older patients with end-stage kidney disease and sarcopenia

**DOI:** 10.1186/s12877-024-05506-z

**Published:** 2024-11-13

**Authors:** Chien-Yao Sun, Lin-Chieh Hsu, Chien-Chou Su, Chung-Yi Li, Chia-Ter Chao, Yu-Tzu Chang, Chia-Ming Chang, Wen-Fong Wang, Wei-Chih Lien

**Affiliations:** 1 Department of Geriatric and Gerontology, National Cheng Kung University Hospital, College of Medicine, National Cheng Kung University, Tainan, Taiwan; 2 Institute of Allied Health Sciences, College of Medicine, National Cheng Kung University, Tainan, Taiwan; 3 Department of Medicine, University of California San Francisco, San Francisco, California USA; 4 Department of Geriatric and Gerontology, College of Medicine, National Cheng Kung University, Tainan, Taiwan; 5 Department of Physical Medicine and Rehabilitation, National Cheng Kung University Hospital, College of Medicine, National Cheng Kung University, Tainan, Taiwan; 6 Clinical Innovation and Research Center, National Cheng Kung University Hospital, College of Medicine, National Cheng Kung University, Tainan, Taiwan; 7 Department of Public Health, College of Medicine, National Cheng Kung University, Tainan, Taiwan; 8 Division of Nephrology, Department of Internal Medicine, National Taiwan University Hospital, Taipei, Taiwan; 9 Division of Nephrology, Department of Internal Medicine, National Taiwan University College of Medicine, Taipei, Taiwan; 10 Graduate Institute of Toxicology, National Taiwan University College of Medicine, Taipei, Taiwan; 11 Division of Nephrology, Department of Internal Medicine, Min Sheng General Hospital, Taoyuan, Taiwan; 12 Graduate Institute of Medical Education and Bioethics, National Taiwan University College of Medicine, Taipei, Taiwan; 13 Department of Internal Medicine, National Cheng Kung University Hospital, College of Medicine, National Cheng Kung University, Tainan, Taiwan; 14 Department of Computer Science and Information Engineering, National Yunlin University of Science and Technology, Yunlin, Taiwan; 15 Department of Physical Medicine and Rehabilitation, College of Medicine, National Cheng Kung University, Tainan, Taiwan

**Keywords:** Triaxial accelerometry, Fall, Hemodialysis, Older, Sarcopenia

## Abstract

**Background:**

Sarcopenia, gait disturbance, and intradialytic hypotension are among the various factors that contribute to fall risk. This study aimed to investigate the relationship between risk of sarcopenia, hemodialysis (HD) session, and long-term fall risk in older end-stage kidney disease (ESKD) patients by analyzing their spatiotemporal gait characteristics.

**Methods:**

We recruited 22 non-demented patients aged ≥ 65 years who were undergoing maintenance HD. Participants were divided into two groups based on their SARC-F score (< 4 and ≥ 4) to identify those with higher and lower risk of sarcopenia. Demographics, comorbidities, and renal parameters were compared between groups. Inertial measurement unit-based technology equipped with triaxial accelerometry and gyroscope was used to evaluate gait characteristics. The gait task was assessed both before and after dialysis using the Timed-Up and Go (TUG) test and a 10-meter walking test at a regular pace. Essential gait parameters were thoroughly analyzed, including gait speed, stride time, stride length, double-support phase, stability, and symmetry. We investigated the interaction between the dialysis procedure and gait components. Outcome of interest was any occurrence of injurious fall during follow-up period. Logistic regression models were employed to examine the relationship between baseline gait markers and long-term fall risk.

**Results:**

The SARC-F ≥ 4 group showed various gait abnormalities, including longer TUG time, slower gait speed, longer stride time, shorter stride length, and longer double support time compared to counterpart (SARC-F < 4). After HD sessions, the SARC-F ≥ 4 group showed a 2.0-second decrease in TUG task time, an 8.0 cm/s increase in gait speed, an 11.6% lower stride time, and a 2.4% increase in gait symmetry with significant group-time interactions. Shorter stride length and longer double support time were associated with injurious falls during the two-year follow-up.

**Conclusion:**

Our study demonstrated the utility of triaxial accelerometers in extracting gait characteristics in older HD patients. High-risk sarcopenia (SARC-F ≥ 4) was associated with various gait abnormalities, some of which partially improved after HD sessions. These gait abnormalities were predictive of future falls, highlighting their prognostic significance.

**Supplementary Information:**

The online version contains supplementary material available at 10.1186/s12877-024-05506-z.

## Background

Aging presents a global challenge that particularly affects people with end-stage kidney disease (ESKD). Registry data from the Taiwan National Health Insurance Research Database demonstrate a steady increase in older individuals undergoing dialysis in the past two decades, particularly over 65 years with ESKD incidence rising from 11,000 to 15,000 per million [1]. In 2020, approximately 345,174 individuals aged ≥ 65 years received renal replacement therapy in the United States (US) [2]. The majority of these older patients receiving renal replacement therapy underwent hemodialysis (HD) instead of peritoneal dialysis (PD) or kidney transplantation (KT). For instance, in 2020, in the US, 77.9% of patients aged ≥ 75 years, 56.1% aged 45–64 years, and 63.2% aged 65–74 years underwent HD [2]. Geriatric syndromes, such as falls, are relatively common in patients with ESKD. A dialysis cohort involving 101,304 HD patients in Taiwan identified 6,286 serious fall using code clustering diagnosis (ICD-9-CM codes: 800–904, 910–957, and E code: E880-E889) over a span of 3.88 years, with a notably higher incidence in those over 65 compared to younger (18–64 years) counterpart (19.32–31.40, and 4.66–10.05 per 1,000 patient-years, respectively) [3]. Falls in older individuals with ESKD are associated with poor outcomes, including fractures, disability [4], long-term institutional care [5], and significant financial burden [6]. Strong evidence suggests that interventions focused on muscle strengthening are effective in reducing the risk of falls in older adults by 12–34% [7, 8]. Given the organ-wide effects on the muscular system, including strength and mass, compounded by accelerated aging in other systemic effects such as bone mineralization and impaired cardiopulmonary fitness, secondary sarcopenia induced by uremia likely plays an intrinsic role in falls [9]. Nevertheless, few studies have specifically investigated the impact of sarcopenia on gait performance, particularly in older adults undergoing HD [10].

Abnormalities in gait parameters, such as slow gait speed, lower extremity dysfunction, and decreased gait performance, have been linked to an increased risk of falls in older adults [11]. In people with non-dialysis chronic kidney disease (CKD), spatiotemporal characteristics of gait phenotype, such as short step, reduced gait stability, and/or marked postural sway, can indicate gait abnormalities before slow gait speed is observed [12]. In addition, uniaxial accelerometer data conducted by Zannoto et al. from patients undergoing HD showed impaired gait performance during lower daily steps and sit-to-stand transitions [10, 13]. Gait is a complex motor function involving multiple domains reflecting the activities of the neurologic, cardiopulmonary, and musculoskeletal systems. Risk factors for abnormal gait are prevalent in the older dialysis population, including cerebrovascular disease, neurologic complications of kidney disease, polypharmacy, and sarcopenia [14]. Most studies focus on simple metrics such as gait speed and do not incorporate more specific temporal and spatial variable measures, while anecdotal evidence assesses subclinical gait features in patients with CKD. A prospective CKD cohort (N: 134, age 81 ± 7 years, estimated glomerular filtration rate (eGFR) 45 ± 11 mL/min/1.73 m^2^) spanning 22.9 months found that gait phenotype, composed of short steps and loss of coordination, was associated with a 72% increased risk of falls, independent of neuropathy, polypharmacy, and gait speed [12]. Studies on gait in patients with ESKD are limited by small sample sizes, cross-sectional designs, and mainly focus on dysregulated posture control after dialysis [15, 16]. The effect of a single HD session on gait performance may also be unique to ESKD. Several small-scale studies have reported decline in lower extremity strength measures [17], 15.2% longer time to complete the sit-to-walk task (*N* = 6) [18], and postural alterations (across position- and velocity-based variables by motion trajectories) after HD sessions (*N* = 12) [19], indicating a controversial but mildly negative impact on gait performance. However, the identification of gait abnormalities in at-risk older patients with ESKD remains undetermined. Our research focused on investigating the short-term effect of high-risk sarcopenia on spatiotemporal gait characteristics after each HD session, while also exploring the long-term relationship between gait markers and fall risk in older dialysis patients.

## Methods

### Study design and participants

**Fig. 1:**
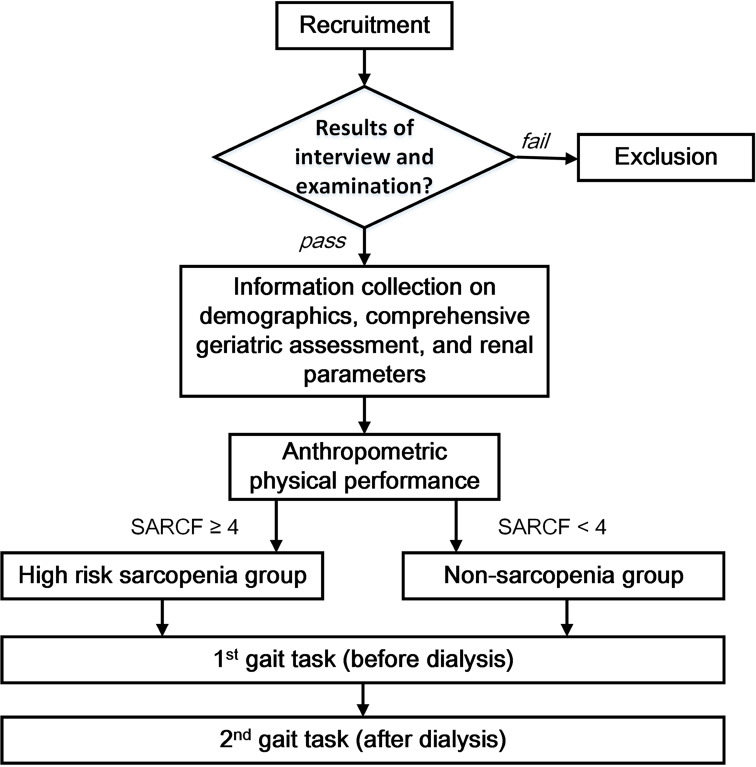
Schematic representation of gait evaluation in older HD patients. SARC-F, strength, ambulation, rising from a chair, stair climbing and history of falling; HD, hemodialysis

We conducted a prospective cohort study in an outpatient HD facility at a university-based medical center between May and September 2020. We used the specific algorithm illustrated in Fig. 1 to recruit participants. Eligible subjects were ambulatory adults aged ≥ 65 years who had been receiving HD for > 3 months, could communicate, and obey verbal orders during the test. Exclusion criteria included terminal illness or malignancy requiring active treatment, inability to walk due to significant clinical illness such as cardiovascular or respiratory diseases, acute musculoskeletal or neurological disturbances, acute infection, or uncorrected visual impairment.

All participants underwent face-to-face interviews and physical examinations. An experienced research nurse collected the demographic characteristics, past medical history, and clinical and mobility assessments. Gait parameters were measured before and 30 min after the HD session on the same day as the basic assessment. All participants wore a wearable device consisting of two triaxial accelerometers (model: LIS3DH, STMicroelectronics) [20] to record the kinematic data of the bilateral lower limbs during walking. Data were transmitted to a research laptop via Bluetooth for further analysis. The study protocol was in accordance with the principles of the Declaration of Helsinki. All eligible participants or their responsible caregivers were fully informed and provided written consent before study participation. The Institutional Review Board of university-based medical center approved the study protocol (A-BR-108-065-T).

### Clinical assessment

We assessed the demographic information of each participant, including age, sex, education level and dialysis vintage, using structured medical questionnaires. We also collected medication and comorbidity information through the electronic medical record (EMR) at the university-based medical center at enrollment. We calculated the total volume of physical activities over the previous month in metabolic equivalent (MET) min/week using the short form International Physical Activity Questionnaire (IPAQ) [21]. We recorded the type of mobility aid and comorbid conditions such as diabetes, parkinsonism, cardiovascular disease, stroke with motor deficits, and medication use such as sedative agents, and alpha-blockers, which were relevant to gait abnormalities.

### Sarcopenia risk assessment using SARC-F

At enrollment, the participants completed the SARC-F (strength, ambulation, rising from a chair, stair climbing and history of falling) questionnaire, which assesses strength, mobility, and falls in the past year. Each item has three levels of severity, with scores ranging from zero to two points. A higher score indicates a greater risk of sarcopenia. A SARC-F score of ≥ 4 out of 10 is recommended in international guidelines to identify individuals at risk of sarcopenia [22]. The SARC-F questionnaire has been validated in different languages in various Asian studies [23] and has been linked to adverse clinical outcomes in older adults, such as reduced quality of life, physical performance, healthcare utilization, and higher mortality rate in the community [22]. In our study, participants were classified into two groups based on their SARC-F scores: high-risk sarcopenia (SARC-F ≥ 4) group and low-risk sarcopenia (SARC-F < 4) group.

### Geriatric assessment of anthropometry and physical performance

Our study included a comprehensive assessment of frailty status using the Fried phenotype (FP), which consists of five components: shrinkage, exhaustion, weakness, slowness, and low activity. These are categorized as robust (0 component), prefrail (1 or 2 components) and frail (3 or more) [24]. Additionally, we collected anthropometric data such as body mass index (BMI), trunk center-of-gravity height, sub-ischial leg length values, and calf circumference (CC). CC was measured using a nonelastic tape around the thickest part of both calves [22], which has been shown to be associated with disability [22]. Low CC is defined as less than 34 cm for men and less than 33 cm for women [22]. By incorporating CC measurements into the SARC-F questionnaire scores, we derived the SARC-CalF (SARC-F combined with calf circumference) with a score of 11 indicating high risk of sarcopenia [22].

The evaluation of physical performance encompassed muscle strength in upper and lower extremities, as well as capacity for balance control. Isometric handgrip strength (HGS) was measured using an electronic hand dynamometer, and the maximum value over three voluntary contraction trials was recorded [25]. Low HGS is defined as less than 28 kg for men and less than 18 kg for women [22]. The extensor and flexor muscle strengths of the lower extremity over the hip, knee, and ankle joints were rated into 5/6 grades (0, 1, 2, 3, 4, and 5) using manual muscle testing while participants were seated in an adjustable straight-backed chair with verbal encouragement to obtain the maximal score. We also assessed balance status using the Berg Balance Scale (BBS), which incorporates 14 balance tasks such as the repeated chair-stand test and unipedal stance time, with total points ranging from 0 to 56 (higher score indicating better performance) [26]. BBS has been shown to be reliable and valid in predicting functional decline, fall occurrence, and healthcare utilization among older patients. Balance is considered impaired for scores below 45 and better for scores of 45 or above [27].

### Renal parameters

The study participants underwent thrice-weekly HD sessions for four hours each time, using a non-reused polysulfone dialyzer (Fresenius Medical Care, Bad Homburg, Germany) and the Fresenius Medical Care 4008 S machine (Bad Homburg, Germany). Throughout the procedure, hemodynamic status, including heart rate and blood pressure, was monitored hourly using a sphygmomanometer equipped with an HD machine. To evaluate dialysis adequacy, the urea reduction rate (URR) was calculated. Other relevant dialysis prescription variables, such as ultrafiltration volume was also obtained. Daily protein intake was estimated using the protein catabolic rate (nPCR) adjusted to the actual body weight based on urea kinetic modeling [28]. Intradialytic hypotension was defined as a drop in systolic blood pressure (SBP) > 20 mmHg or nadir < 90 mm Hg, or symptomatic hypotension episode requiring intervention, such as fluid challenge or dialysis cessation. Serum concentrations of hemoglobin, albumin, calcium, phosphorus, sodium, potassium, C-reactive protein (CRP), β2-microglobulin, intact parathyroid hormone (iPTH), and total carbon dioxide were measured using a Roche Cobas 8000 Analyzer (Roche Diagnostics, Germany) in a single laboratory (Laboratory of Clinical Chemistry, NCKUH).

### Gait task

**Fig. 2:**
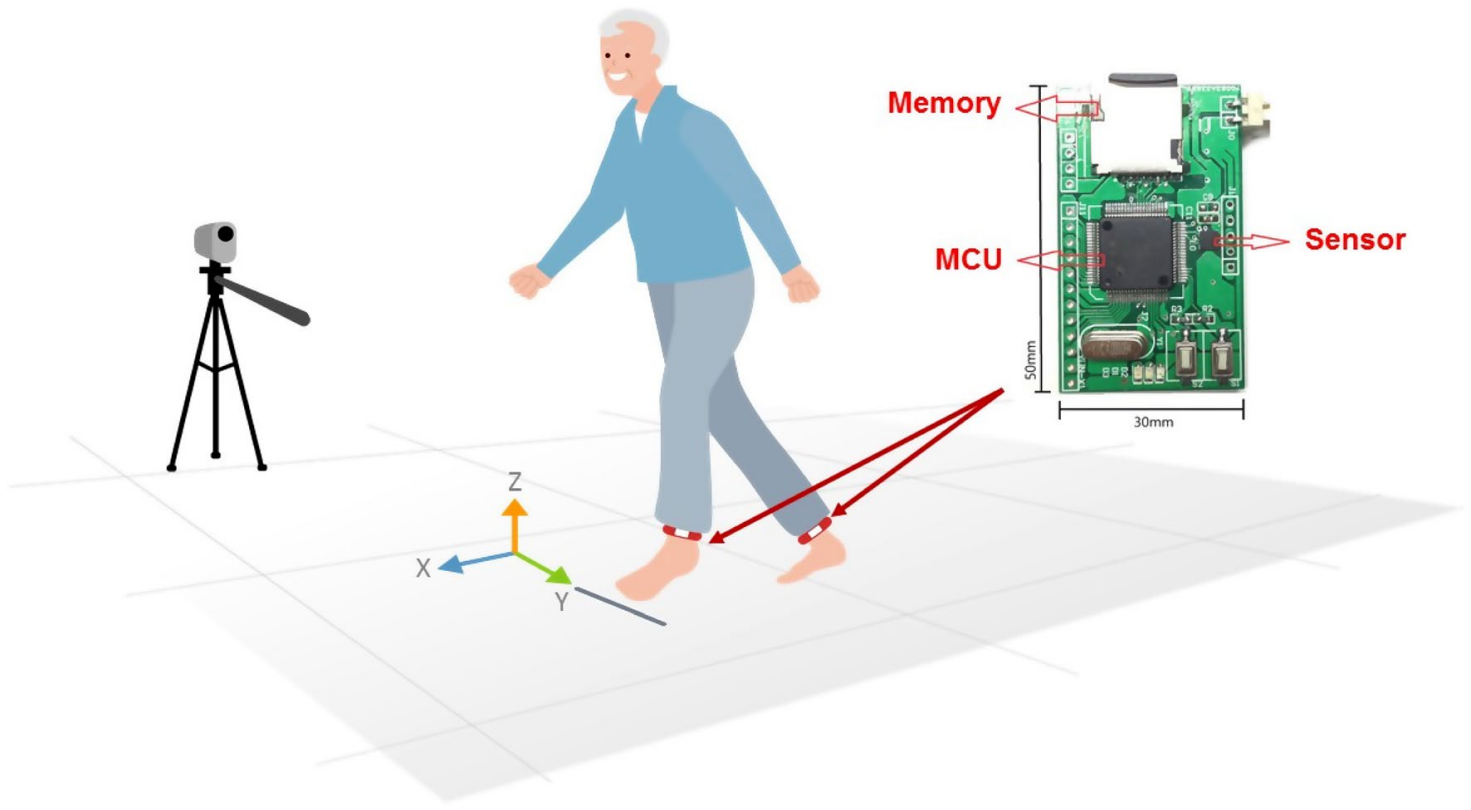
Diagram illustration of procedure for signal conditioning and example of gyroscope and accelerometer signals of a sample subject (IMU located at ankle). IMU, inertial measurement unit; MCU, microcontroller unit

**Fig. 3:**
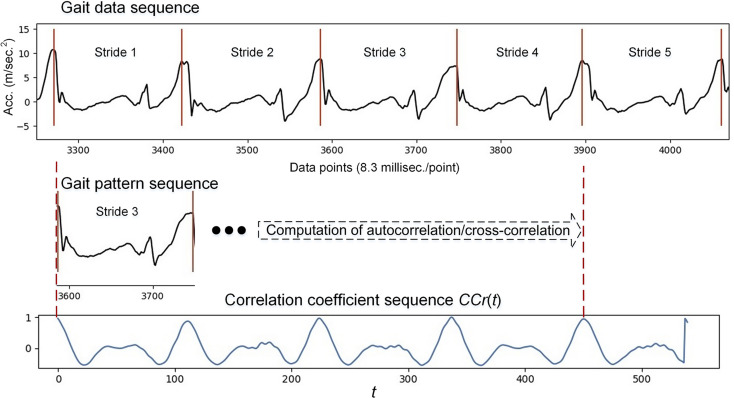
Example of acceleration (acc) measured by the accelerometer of a sample subject from 5 strides of left lower extremity

Detailed descriptions of how to acquire and process gait data to derive each temporal parameter were provided in our previous work [29, 30]. The gait measurement device utilized in this study was developed in our laboratory (Fig. 2). The device incorporates a triaxial accelerometer (model: LIS3DH, STMicroelectronics) [20]. Two wearable devices were affixed to each side of the participant’s malleolus, and gait data were recorded during the five intermediate steps of each gait trial at a sampling rate of 120 Hz, enabling the acquisition of quantitative gait parameters. Accelerometers are used to capture kinematic data along three axes (vertical, anterior-posterior, and mid-lateral) and have been validated to measure quantitative gait parameters with high reliability in various clinical settings [17, 18, 29, 30]. The examples of the accelerometer signals were shown in Fig. 3. After a rest period of approximately 30 min, participants completed the Timed-Up and Go (TUG) task and the 10-meter walk test (10MWT) in both the pre- and post-HD trials. Participants were instructed to walk at their normal pace in a well-lit hallway, and gait parameters were calculated based on the mean of two trials. Well-trained research staff, blinded to the group allocation and study objectives, manually processed the gait data. The gait cycle is the time between two successive initial contacts of the same limb with the ground and includes the swing and stance phases. The double-support phase is an important subset of the stance phase, which is related to balance control. The spatial and temporal gait parameters analyzed in this study included TUG task time, gait speed, stride length, stride time, stride time variability, stride length, cadence, double support time and regularity of acceleration signals, such as gait symmetry (cross-correlation of contralateral acceleration in the corresponding gait cycle in the bilateral lower limbs [31]) and gait stability (autocorrelation of ipsilateral acceleration in two consecutive gait cycles in the dominant lower limbs [31]).

The TUG task measures the amount of time it takes a person to get up from an unarmed chair, walk three meters, turn around, and return to the chair. The European Working Group on Sarcopenia in Older People (EWGSOP) criteria recommend it as a measure of physical performance. Gait speed, measured using the 10MWT in accordance with international consensus recommendations, is highly predictive of adverse health outcomes, such as disability and mortality [22]. Stride length was calculated by multiplying the gait speed by two and dividing by the cadence number, which was obtained by counting the number of steps during the 10MWT and dividing by the observed time period. The duration of the gait cycle (i.e., stride time) is the amount of time elapsed between successive heel contacts of the same foot. Stride time variability was expressed as a percentage of the variation from the average stride time. The regularity of acceleration signals can be used to predict the occurrence of harmful falls and to quantify the improvement of gait measures with exercise and drug interventions [30].

To obtain a cross-correlation coefficient list for evaluating gait symmetry and stability [32], a gait data sequence, which is the filtered gait data obtained in one gait trial, and a gait pattern sequence, which is one complete gait cycle selected from the gait data sequence, are necessary (Fig. 3). Let *q*_*i*_ and *p*_*i*_ be the gait data sequence and gait pattern sequence, respectively, where *i* is the *i*-th element of the sequences. Suppose the length of *q* is *m* and *p* is *n*., where *m* ≥ *n*. Subsequently, the computation of the correlation coefficients for the gait symmetry and stability can be performed using Eq. (1) below.

1 $$\:CCr\left(t\right)=\frac{\sum\:_{i=1}^{n}({p}_{i}-\stackrel{-}{p})({q}_{i+t}-{\stackrel{-}{q}}_{t})}{\sqrt{{\sum\:}_{i=1}^{n}{({p}_{i}-\stackrel{-}{p})}^{2}\sum\:_{i=1}^{n}{({q}_{i+t}-{\stackrel{-}{q}}_{t})}^{2}}}$$

Where *t* is an integer shift variable ranging from *0* to *m–n*, and $\:\stackrel{-}{p}$ and $\:\stackrel{-}{{q}_{t}}$ are the means of the *p* sequence (*p*_1_, …, *p*_n_) and *q* partial sequence (*q*_(*t*+1)_, …, *q*_(*t*+n)_), respectively. For each value of CCr(*t*), the correlation coefficient varied from − 1 to 1, and an example of the correlation coefficient sequence *CCr*(*t*) can be found in Fig. 3.

### Outcome

The study followed participants until death, transfer to a non-study dialysis center, or January 31, 2023. Every month, researchers interviewed participants either in-person or via phone using a standard questionnaire to identify injurious falls. Injurious falls were defined as unintentional falls from one level to a lower height, excluding significant intrinsic events like syncope or seizures, as well as extrinsic events such as traffic accidents [33]. This definition is consistent with that used in other large randomized trials [34]. These falls were associated with adverse events such as injuries (contusions, lacerations, and fractures), emergency department visits, or hospitalizations. Fall outcomes were classified as incident (first fall in participants with no history of falls), recurrent (any fall in participants with a history of falls), single (one fall event during follow-up), or multiple (more than one fall during follow-up).

### Statistical analysis

The study presented numerical data as mean values with standard deviations for normal distribution, median values with interquartile ranges for skewed distribution, and numbers and percentages for categorical data. Baseline characteristics, including demographic, clinical, laboratory, mobile, and gait data, were compared between the groups using appropriate nonparametric tests. Quantitative variables were analyzed using Student’s t-test or Wilcoxon–Mann–Whitney test, while qualitative variables were analyzed using χ2 or Fisher’s exact test. Generalized estimating equations (GEE) were used to analyze variations in gait parameters across different sarcopenia risk groups and their interactions with HD sessions [35]. We also applied GEE analysis to other geriatric parameters (HGS, GS, CC, SARC-CalF, BBS, FP) and examined their temporal interaction with gait markers. Logistic regression analysis was performed to examine the possible association between spatiotemporal gait parameters and the risk of injurious falls during follow-up in older patients with ESKD. Odds ratios (OR) and 95% confidence intervals (CI) were estimated using these models. To reduce potential confounding effects, our multivariate logistic regression models included variables such as age and sex, given their known associations with our study outcomes [36, 37]. Analyses were reiterated for SARC-F and other geriatric parameters (HGS, CC, SARC-CalF, BBS) in relation to longitudinal fall risk. We calculated sensitivity and specificity for the binary longitudinal fall risk in relation to SARC-F and four selected reference geriatric parameters/instruments (HGS, CC, SARC-CalF, BBS). All statistical analyses were performed using SPSS 22.0 for Windows (SPSS Inc., IBM Company, Chicago, IL), and graphs were plotted using GraphPad Prism version 5.01 (GraphPad Software, Inc., La Jolla, CA). The significance level was set at a two-sided *p*-value < 0.05.

## Results

### Participant characteristics

**Fig. 4:**
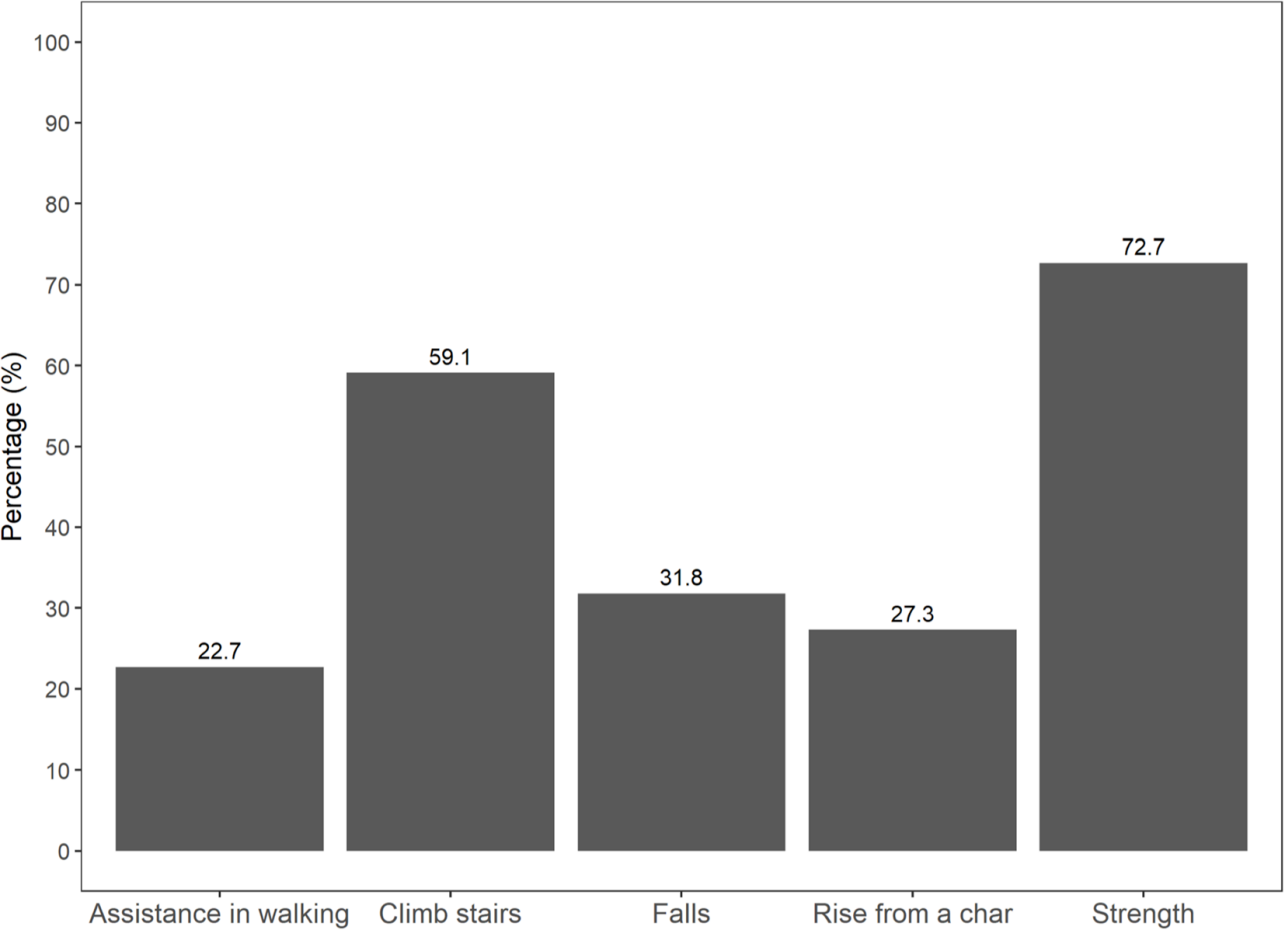
Prevalence of SARC-F components in older HD patients. SARC-F, strength, ambulation, rising from a chair, stair climbing and history of falling; HD, hemodialysis

In total, 34 participants were enrolled in the study, and 22 patients completed pre- and post- HD gait task evaluations. Of these, nine participants were classified as high-risk sarcopenia group, and the remaining 13 participants were in the low-risk group. Among the five components of SARC-F, low strength was the most common (72.7%), followed by difficulty climbing stairs (59.1%) and previous falls (31.8%) (Fig. 4). Half of the participants were women, and the median age was 73.7 years (interquartile range (IQR) 68.3–78.0 years). Participants had a dialysis vintage of 4.5 years, optimal dialysis dose prescription (URR: 78.2%) and were well-nourished (estimated dietary protein intake (nPCR) 1.1 g/kg/d, median BMI 23.5 kg/m^2^, serum albumin level 4.2 g/dL) with a low occurrence of intradialytic hypotension (9.1%). However, geriatric comorbidities were common in this cohort, such as polypharmacy (medication count ≥ 10: 100%), walking aid use in occasional daily activities (27.3%), and low physical activity (median, 594 MET-min/week).

Table 1 summarizes the patients’ baseline demographic and clinical characteristics stratified by SARC-F status. The difference between the two groups regarding basic data, comorbid profile, and renal parameter, was not marked. Those with a SARC-F score of 4 or higher had slightly higher serum calcium levels compared to those with a SARC-F score less than 4. Participants with SARC-F ≥ 4 had shorter height of center gravity, leg length, and slightly smaller CC compared to the counterpart. The high-risk sarcopenia group also demonstrated higher SARC-CalF scores and poorer physical function, including lower handgrip strength (HGS), reduced Berg Balance Scale (BBS) scores (Table 1).

**Table 1:** Baseline characteristics of the older hemodialysis patients and comparisons between different risk of sarcopenia groups

	All (*N* = 22)	Sarcopenia risk (SARC-F)		*P* value
		High-risk ^a^ (*N* = 9)	Low-risk ^a^ (*N* = 13)	
**Baseline characteristics**				
Age (years)	73.7 (68.3–79.4)	74.0 (71.1–84.0)	69.0 (67.0–78.0)	0.186
Women	11 (50.0)	7 (77.8)	4 (30.8)	0.080
Dialysis vintage (year)	4.5 (2.6–5.7)	5.4 (4.0-6.6)	2.7 (2.2–4.6)	0.082
Education level				0.590
≤ Elementary school	15 (68.2)	7 (77.8)	8 (61.5)	
High school	1 (4.5)	0 (0)	1 (7.7)	
College or above	6 (27.3)	2 (22.2)	4 (30.8)	
Physical activity (MET-h/week)	594 (0-693)	462 (0-694)	640 (140–809)	0.393
Walking aid use	6 (27.3)	4 (44.4)	2 (15.4)	0.132
Diabetes mellitus	12 (54.5)	4 (44.4)	8 (61.5)	0.670
Parkinsonism	1 (4.5)	1 (11.1)	0 (0.0)	0.410
Cardiovascular disease	10 (45.5)	3 (33.3)	7 (53.8)	0.420
Stroke with hemiparesis	3 (13.6)	1 (11.1)	2 (15.4)	0.774
Medication count	13.0 (10.0–17.0)	10.0 (10.0–19.0)	14.0 (9.0–16.0)	0.560
Sedative drugs	9 (40.9)	5 (55.6)	4 (20.8)	0.384
Alpha-blocker	4 (18.2)	1 (11.1)	3 (23.1)	0.620
Fried				0.072
Robust	3 (13.6)	0 (0)	3 (23.1)	
Prefrail	8 (36.4)	2 (22.2)	6 (46.2)	
Frail	11 (50.0)	7 (77.8)	4 (30.8)	
SARC-CalF score	10.5 (1.0–14.0)	14.0 (10.5–15.5)	2 (0-11.5)	0.001
SARC-CalF ≥ 11	11 (50.0)	7 (77.8)	4 (30.8)	0.080
**Anthropometry**				
BMI (kg/m^2^)	23.5 (22.7–26.1)	23.2 (22.7–24.3)	25.6 (22.8–27.3)	0.393
Height of center gravity (cm)	91.3 (85.5–96.0)	85.5 (84.3–90.3)	95.0 (89.0-98.5)	0.002
Leg length (cm)	80.0 (75.8–86.3)	76.0 (73.0-77.3)	84.0 (80.0-89.5)	0.001
Calf circumference (cm)	32.3 (30.0-34.9)	30.0 (29.3–33.0)	32.5 (31.5–37.8)	0.050
**Physical Performance**				
Handgrip strength (kg)	21.9 (15.7–28.2)	18.2 (13.5–20.2)	27.2 (20.9–31.2)	< 0.001
Hip flexors (score)	4.0 (4.0–5.0)	4.0 (3.5–4.5)	4.0 (4.0–5.0)	0.186
Knee extensors (score)	4.0 (4.0–5.0)	4.0 (4.0–5.0)	5.0 (4.0–5.0)	0.512
Ankle plantar flexors (score)	4.5 (4.0–5.0)	4.0 (4.0–5.0)	5.0 (4.0–5.0)	0.845
Berg balance score (points)	42.0 (37.3–50.3)	35.0 (23.0-42.5)	47.0 (41.0–52.0)	0.003
**Renal parameters**				
Urea reduction ratio	78.2 (74.8–80.5)	80.3 (76.0-82.9)	77.6 (74.6–79.0)	0.164
Ultrafiltration volume (L)	2.1 (1.1–2.4)	2.1 (0.9–2.4)	2.0 (1-2.35)	0.794
nPCR (g/kg/d)	1.1 (1.0-1.3)	1.1 (0.9–1.3)	1.1 (1.0-1.3)	0.948
Intradialytic hypotension	2 (9.1)	1 (11.1)	1 (7.7)	0.780
Hemoglobin (g/dL)	10.8 (9.9–11.8)	10.8 (9.4–11.1)	10.8 (9.9–11.8)	0.556
Albumin (g/dL)	4.2 (4.0-4.5)	4.0 (3.9–4.5)	4.2 (4.0-4.5)	0.292
Calcium (mg/dL)	9.6 (9.0-9.8)	9.6 (9.0-9.8)	9.1 (8.6–9.2)	0.025
Phosphorus (mg/dL)	4.3 (3.8–5.3)	4.4 (2.8–5.4)	4.3 (3.8–5.3)	0.845
Sodium (mmol/L)	138.0 (136.0-142.5)	137.0 (136.0-139.0)	138.0 (136.0-142.5)	0.512
Potassium (mmol/L)	4.4 (4.1–4.7)	4.6 (4.2–4.9)	4.4 (4.1–4.7)	0.431
CRP (mg/dL)	4.5 (2.4–16.7)	5.7 (2.3–10.8)	4.5 (2.4–16.7)	0.896
β_2_-microglobulin (mg/L)	33.3 (29.2–38.2)	31.0 (29.7–33.5)	33.9 (26.7–43.3)	0.357
iPTH (pg/mL)	270.0 (174.5–502.0)	341.0 (186.5-602.5)	212.0 (121.5-367.5)	0.186
total CO_2_ (mmol/L)	23.5 (23.0-25.3)	23.0 (22.5–25.0)	24.0 (23.0–26.0)	0.556

Values for categorical variables given as number (percentage); values for continuous variables given as median (interquartile range)

Abbreviations: MET, metabolic equivalent; ACEi, angiotensin-converting enzyme inhibitors; ARB, angiotensin II receptor blocker; SARC-CalF, SARC-F combined with calf circumference; BMI, body mass index; nPCR, normalized protein catabolic rate; CRP, C-reactive protein; iPTH, Intact parathyroid hormone; CO_2_, carbon dioxide

^a^ Sarcopenia risk is categorized as high-risk group (SARC-F ≥ 4) and low-risk group (SARC-F < 4)

### Quantitative gait analysis

**Fig. 5:**
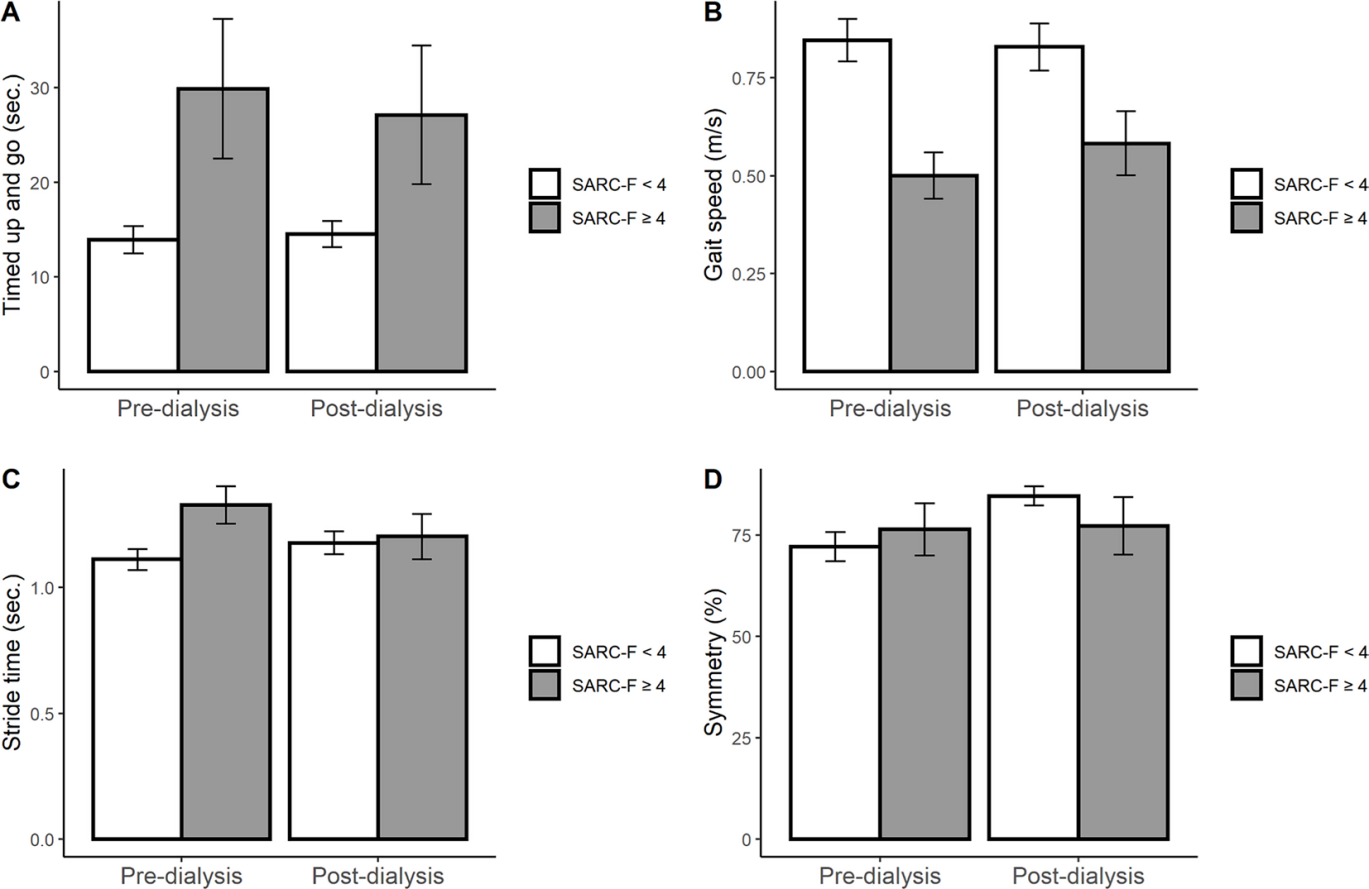
Changes of gait parameters in comparison of pre- and post-HD status between different risk groups of sarcopenia (Dark line indicates SARC-F ≥ 4 group, white line indicates SARC-F < 4 group). (**A**) Timed-Up and Go (interaction *p* < 0.001), (**B**) Gait speed (interaction *p* = 0.003), (**C**) Stride time (interaction *p* = 0.024), (**D**) Symmetry (interaction *p* = 0.005). HD, hemodialysis; SARC-F, strength, ambulation, rising from a chair, stair climbing and history of falling

Table 2 presents the differences in several quantitative gait markers between the two groups. Compared to their counterparts, individuals in high-risk sarcopenia group took longer to complete the TUG task, had slower gait speeds (GS), shorter stride lengths (SL), spent longer stride time (ST), and double support (DS) time. These quantitative differences in gait markers persisted in the post-HD trials, except for stride time. Changes in gait characteristics were also observed in the groups divided by HGS, BBS, GS and FP, but not in those divided by CC and SARC-CalF scoring (Supplementary Tables 1–6), with SARC-F showing the most significant differences (Table 2). To investigate the impact of HD sessions on gait characteristics, we added interaction terms to explore the association between pre- and post-HD sessions and the trend of gait changes (Table 2). Several changes were revealed in gait characteristics in the SARC-F ≥ 4 group after HD sessions, including a 2.0-second decrease in TUG task time (interaction *p* < 0.001), a 0.08 m/s increase in gait speed (interaction *p* = 0.003), a 0.16-second lower stride time (interaction *p* = 0.024), and a 2.4% increase in gait symmetry (interaction *p* = 0.005) compared to those with SARC-F < 4 (Fig. 5).

**Table 2:** Analysis of gait parameters in different sarcopenic risk groups between pre- and post-HD sessions

	Sarcopenia risk (SARC-F)				
	High-risk ^a^ (*N* = 9)		Low-risk ^a^ (*N* = 13)		
Time Gait parameters	Pre-dialysis	Post-dialysis	Pre-dialysis	Post-dialysis	Time x Sarcopenia risk Interaction *p* value
TUG time (sec)	21.0 (18.3–35.3) ^*^	19.0 (18.0-24.3) ^†^	12.0 (11.0-16.8) ^*^	12.5 (10.8–17.8) ^†^	< 0.001
Gait speed (m/s)	0.52 (0.44–0.62) ^*^	0.60 (0.41–0.72) ^†^	0.94 (0.67-1.00) ^*^	0.88(0.66–0.99) ^†^	0.003
Stride time (sec)	1.38 (1.19–1.51) ^*^	1.22 (0.94–1.46)	1.08 (1.06–1.16) ^*^	1.20 (1.06–1.31)	0.024
Stride time variability (%)	12.3 (8.7–34.9)	14.0 (11.0-34.1)	14.5 (9.5–25.8)	17.0 (13.5–20.1)	0.496
Stride length (m)	0.76 (0.45–0.81) ^*^	0.67 (0.53–0.87) ^†^	1.0 (0.83–1.09) ^*^	0.99 (0.78–1.13) ^†^	0.718
Cadence (steps/min)	95.0 (81.0-118.5)	105.0 (88.0-124.0)	111.0 (99.5–117.0)	104.0 (94.5–114.0)	0.270
Double support (sec)	0.39 (0.33–0.43) ^*^	0.34 (0.31–0.48)	0.33 (0.31–0.36) ^*^	0.32 (0.30–0.37)	0.830
Double support (%)	29.9 (29.3–30.3)	30.0 (29.4–30.2)	30.2 (29.6–30.3)	29.8 (29.6–30.2)	0.111
Stability (%)	76.8 (72.7–82.4) ^*^	78.3 (69.2–84.3)	64.0 (57.0-73.2) ^*^	78.7 (66.0-85.7)	0.060
Symmetry (%)	81.5 (68.9–89.8)	83.9 (69.4–90.8)	66.5 (61.7–84.8)	85.0 (77.4–92.2)	0.005

Values for continuous variables given as median (interquartile range)

Abbreviation: TUG, Timed-Up and Go

^a^ Sarcopenia risk is categorized as high-risk gorup (SARC-F ≥ 4) and low-risk gorup (SARC-F < 4)

*p < 0.05, comparison between high-risk sarcopenia group and low-risk sarcopenia group in pre-dialysis

†*p* < 0.05, comparison between high-risk sarcopenia group and low-risk sarcopenia group in post-dialysis

### Gait parameters and the risk of falls

During the 2-year follow-up period, 22 patients experienced 10 injurious falls (45.5% of cohort cases) and three unfortunate deaths (Supplementary Table 7). Patients in the SARC-F ≥ 4 group had a higher incidence of falls compared to their counterparts (77.8% vs. 23.1%, *p* = 0.027). During the observation period of 365.3 patient-months, there were seven fall incidences, corresponding to 19.2 per 1000 patient-months. Of the six patients who had a history of falls before entering the cohort, 50% (*N* = 3) experienced recurrent falls. Single falls accounted for 60% of the cases, while 40% of patients fell two or more times.

Our analysis revealed a significant association between high-risk sarcopenia and other geriatric parameters with the risk of injurious falls. The OR for the high-risk groups of SARC-F, HGS, and BBS were 7.36, 10.42, and 25.53, respectively (Supplementary Table 8). However, similar associations were not observed with CC and SARC-CalF (Supplementary Table 8). Longer TUG task time per 1-second increase (OR 1.50, 95% CI 1.05–2.15), lower gait speed per 0.1 m/s decrease (OR 7.32, 95% CI 1.13–47.50), were associated with injurious falls (Table 3). Additionally, the point estimate for shorter stride length per 10 cm decrease (OR 5.51, 95% CI 0.92–32.85), and greater double support per 1 SD increase (OR 2.82, 95% CI 0.73–10.91) remained large with borderline statistical significance after adjustment for age and sex variables (Table 3).

**Table 3:** Association between gait parameters and risk for injurious falls

Gait parameters	Unit change	Median (IQR)	Variable	Odds ratio	*p* value
Pre-HD					
TUG time (sec)	1 s increase	16.8 (11.4–22.5)	Univariable	1.44 (1.07–1.94)	0.016
			Multivariable*	1.50 (1.05–2.15)	0.028
Gait speed (m/sec)	0.1 m/s decrease	0.70 (0.52–0.96)	Univariable	5.13 (1.23–21.48)	0.025
			Multivariable	7.32 (1.13–47.50)	0.037
Stride time (sec)	per 1 SD increase	1.14 (1.06–1.40)	Univariable	2.62 (0.91–7.58)	0.076
			Multivariable*	2.35 (0.76–7.31)	0.139
Stride length (meter)	10 cm decrease	0.82 (0.72–1.01)	Univariable	3.25 (1.16–9.12)	0.025
			Multivariable*	5.51 (0.92–32.85)	0.061
Cadence (steps/min)	10-step decrease	108.0 (91.5–113.0)	Univariable	1.58 (0.85–2.92)	0.148
			Multivariable*	1.48 (0.77–2.85)	0.234
Double support (sec)	per 1 SD increase	0.34 (0.32–0.39)	Univariable	3.58 (1.03–12.52)	0.045
			Multivariable*	2.82 (0.73–10.91)	0.134
Double support (%)	10% increase	30.0 (29.6–30.2)	Univariable	< 0.01(< 0.01–34.52)	0.086
			Multivariable*	< 0.01 (< 0.01-102.84)	0.083
Stride time variability (%)	10% increase	14.0 (8.7–26.9)	Univariable	1.06 (0.81–1.40)	0.660
			Multivariable*	1.09 (0.79–1.51)	0.606
Stability (%)	10% decrease	71.1 (61.2–78.1)	Univariable	0.87 (0.48–1.60)	0.658
			Multivariable*	0.98 (0.51–1.89)	0.943
Symmetry (%)	10% decrease	78.5 (62.1–86.0)	Univariable	0.79 (0.44–1.42)	0.421
			Multivariable*	0.85 (0.46–1.56)	0.590
Post-HD					
TUG time (sec)	1 s increase	17.0 (12.0-22.3)	Univariable	1.55 (1.09–2.20)	0.015
			Multivariable*	1.57 (1.06–2.33)	0.025
Gait speed (m/sec)	0.1 m/s decrease	0.71 (0.56–0.93)	Univariable	4.26 (1.25–14.58)	0.021
			Multivariable*	7.28 (0.87–60.70)	0.067
Stride time (sec)	per 1 SD increase	1.21 (1.06–1.33)	Univariable	2.22 (0.81–6.09)	0.123
			Multivariable*	1.79 (0.58–5.56)	0.315
Stride length (meter)	10 cm decrease	0.84 (0.65–1.04)	Univariable	2.30 (1.20–4.39)	0.012
			Multivariable*	6.89 (0.99–48.17)	0.052
Cadence (steps/min)	10-step decrease	99.0 (91.5-113.5)	Univariable	1.69 (0.91–3.16)	0.100
			Multivariable*	1.47 (0.73–2.97)	0.280
Double support (sec)	per 1 SD increase	0.34 (0.30–0.39)	Univariable	1.22 (0.51–2.92)	0.649
			Multivariable*	1.14 (0.96–1.36)	0.138
Double support (%)	10% increase	29.9 (29.6–30.2)	Univariable	0.30 (0.02–4.62)	0.387
			Multivariable*	0.20 (0.02–2.40)	0.206
Stride time variability (%)	10% increase	15.3 (12.6–20.0)	Univariable	1.31 (0.70–2.45)	0.395
			Multivariable*	1.34 (0.64–2.81)	0.446
Stability (%)	10% decrease	78.4 (69.5–84.1)	Univariable	1.10 (0.67–1.81)	0.704
			Multivariable*	1.06 (0.63–1.80)	0.818
Symmetry (%)	10% decrease	85.0 (77.5–91.4)	Univariable	1.26 (0.68–2.33)	0.466
			Multivariable*	1.24 (0.61–2.49)	0.556

Abbreviations: IQR, interquartile range; HD, hemodialysis; TUG, Timed-Up and Go; SD, standard deviation

* Adjusted for age, sex

## Discussion

Our study is the first to prospectively examine the relationship between quantitative gait data obtained using a triaxial accelerometer and fall risk in dialysis patients. We found that older HD patients in high-risk sarcopenia group, as identified by SARC-F criteria, had disturbed spatiotemporal gait characteristics, including shorter steps and altered time spent in the gait cycle. We observed positive interactions between dialysis sessions and gait parameters, including improved TUG and gait speed, shorter stride time, and improved acceleration signal regularity, such as gait symmetry. Furthermore, abnormal gait parameters predicted the risk of falling within 2 years. These findings suggest that a gait-assessing tool could be recommended as part of the care component for older HD patients, helping to identify those at risk and enabling interventions to prevent fall-related injuries.

Patients with ESKD have slower gait velocity than non-dialysis patients with CKD across various stages, regardless of self-selected or maximal speed [38]. Global speed is influenced by interconnected gait domains. For instance, spending more time in the double stance can result in a decrease in step length owing to a reduced swing phase [39]. Lower eGFR and higher albuminuria are linked to worse gait velocity and several gait domains, including rhythm, phases, pace, and base of support [12, 40]. Our study found that patients with ESKD who were in high-risk sarcopenia group had gait alterations characterized by more time spent in the TUG, slower gait speed, longer stride time, shorter stride length, and more time in the double support phase compared to the low-risk sarcopenia group. These gait alterations were consistent across pre- and post-HD tests, except for stride time, which resembled the phenotype observed in patients with CKD [12]. Tran et al. also found a linear association between eGFR (per 10 mL/min/1.73 m^2^ decline) and quantitative gait abnormalities (speed − 3.3 cm/s, stride length − 3.6 cm, swing phase − 0.7%, and double support + 1.1%) [12]. Unlike neurological conditions such as stroke, parkinsonism, or dementia [12], our study did not find gait variability or cadence alterations in high-risk sarcopenic patients with ESKD. These abnormalities may be attributed to differences in balance, muscle strength, and uremic solute clearance between the SARC-F groups (Table 1). Larger-scale studies are required to confirm sarcopenia-related gait features in patients with ESKD.

Muscle mass and function are important determinants of outcomes in patients with CKD, although their impacts appear to vary. The ACTIVE/ADIPOSE study demonstrated that grip weakness and gait slowness significantly improved mortality prediction (C-statistic increased from 0.63 to 0.68) and improved net reclassification indices more effectively than muscle mass measures [41]. In our study, we found that incorporating calf circumference into the SARC-F score to form SARC-CalF did not improve the predictive accuracy for fall risk (Supplementary Table 8). This result contrasts with previous findings suggesting that SARC-CalF generally provides better screening accuracy for sarcopenia in CKD patients than SARC-F alone [42, 43]. The reasons for this discrepancy are multifaceted. Our primary outcome of interest was fall prediction rather than sarcopenia diagnosis. For long-term outcomes such as mortality, SARC-F has been shown to have non-inferior predictive efficacy compared to SARC-CalF. A study involving elderly nursing home residents identified SARC-F as independently associated with an increased risk of one-year mortality, while SARC-CalF was not [44]. Common techniques for assessing body composition, such as bioelectrical impedance analysis (BIA) and dual-energy X-ray absorptiometry (DXA), measure total body water and bone mineral content in the former, and lean body mass in the latter [45]. Both methods assume a fixed fractional hydration ratio of 73% [46], which may not hold true in end-stage kidney disease (ESKD) due to fluid overload or edema [47]. In ESKD patients, uremia may lead to muscle system alterations, such as myosteatosis and sarcopenic obesity [14]. These conditions can obscure the readings of crude body composition markers, such as BMI and calf circumference, which may not accurately reflect true lean mass as the general population [22]. In our study, even patients considered well-nourished by BMI standards (an average BMI of 23.2 kg/m² in the SARC-F ≥ 4 group, Table 1) might still be at significant risk for sarcopenia. Given the moderate sensitivity (28.9–55.3%) of SARC-F in screening for sarcopenia [48], our study also illustrates the efficacy of the SARC-F tool in classifying gait variables and its association with long-term outcomes such as the OR of injurious falls in community-based outpatient dialysis, in contrast to the limited efficacy of CC and SARC-CalF. These findings underscore the importance of further research to validate the clinical utility of SARC-F in classifying pathological gait, particularly its sensitivity and specificity for individual gait measures in patients with ESKD.

Previous studies have shown more balance complications in gait tests after HD sessions [17–19], which may be caused by inadequate plasma refilling during ultrafiltration, leading to fatigue, hemodynamic instability, and reduced neuromotor coordination. However, our study found that a single HD session may function as a positive moderator on gait characteristics, particularly in the high-risk sarcopenia group, including improved gait speed, stride time, and symmetry, as shown in Table 2. This may be due to several mechanisms, including the removal of accumulated interstitial fluid more from the peripheral extremities than the trunk [49], a phenomenon that is more pronounced in the high-risk sarcopenia group and can improve pressure transduction in dynamic mechanics, facilitating motor function in the lower limbs [50]. Moreover, correcting acidosis through alkali addition and organic acid disposition during dialysis can improve calcium-dependent actomyosin interactions [51]. Normalization of serum levels of cations (e.g., calcium, potassium, and magnesium) after HD can also stabilize the transmembrane action potential of excitable tissues, resulting in increased resistance to fatigue [52]. Furthermore, prolonged dialysis treatments may improve nerve conduction and partially alleviate axonal degeneration in the legs caused by potential neurotoxins such as myoinositol and PTH [53]. It is worth noting that the intra-dialytic hemodynamics in our study were smooth without dialytic hypotension episodes requiring saline infusion that may affect gait performance. Overall, our study highlights the bidirectional influence of HD therapy on spatiotemporal gait parameters, which may favorably affect post-dialysis gait function.

Our study has shown that using a wearable triaxial accelerometer device to monitor gait patterns continuously can provide qualitative (regularity of acceleration signals, e.g., gait stability and symmetry) and quantitative (e.g., stride length, cadence, and variability) information in dialysis patients. While the widely used gait speed measure may not capture the full spectrum of gait dysfunction in CKD populations, pressure-sensing walkways (e.g., GAITRite system) require more cost, dedicated space and are limited in outdoor settings. The triaxial accelerometer, which uses a gyroscope and inertial measurement unit (IMU)-based technology to measure key components of gait, such as acceleration in three axes and rotation rate [54], is a convenient tool for collecting objective data on subtle gait features. It is also portable, cost-effective, and provides real-time feedback information on gait variables but requires personnel training and time-consuming interpretation. However, studies that have applied IMUs to collect gait data from patients with advanced CKD are mostly small-scale (*N* = 5–14) and cross-sectional, addressing the relationship between gait movement and clinical variables (e.g., residual renal function and impact of dialysis therapy) [15, 17, 18, 55]. Several inconsistencies have been noted in the methodology of detecting spatiotemporal information, patient characteristics, gait task, different gait parameters obtained, and lack of universal outcomes, which may reduce the comparability among studies (Supplementary Table 9). Further work is required for patients with ESKD to establish normalized cut-offs for gait parameters by following the standardized procedures adopted in our study with thorough computerized gait measurements.

Our study indicates that in older HD patients, a shortened stride length may predict the risk of injurious falling over two years, in addition to established measures such as TUG and gait speed. Similar findings have been reported in other prospective cohort studies of community-dwelling older adults [56]. Sedaghat et al. conducted a retrospective study of 1,430 participants with early CKD (stage 1–2) and found an association between worse gait scores (including stride length and six other spatiotemporal gait domains) and falls in the previous year [40]. In a prospective study of 134 individuals with CKD stage 3, Tran et al. also reported that any abnormal gait signs, such as shorter stride length or longer stance phase duration, were associated with a 72% higher adjusted risk of falls [12]. A shorter stride length, among other gait features, may reflect a more cautious gait pattern due to changes in proprioception, muscle power, and flexibility that occur with aging and kidney disease. This pattern may require a greater number of steps to cover the same distance and could increase the risk of tripping, balance loss, or falling. Our study emphasizes the importance of specific gait components in routine care for patients with ESKD, which can enable targeted interventions aimed at reducing the risk of falls [57].

Our findings demonstrate differential effects of HD on gait parameters, where improvements in stride time, gait speed, and symmetry post-HD did not translate to changes in parameters closely linked to fall risk, such as stride length and double support time. This discrepancy can be attributed to the physiological dynamics unique to HD. First, although hemodynamics remained stable in our cohort during a single hemodialysis session, the study did not investigate long-term subtle fluctuations in cerebral blood flow [58], which could influence balance-related gait components like double support time. Second, gait features such as stride length, associated with fall risk, may better reflect changes in cognitive, proprioceptive, or central nervous system processes, rather than being immediately responsive to acute effects of HD. Shorter stride lengths serve as early signs of cognitive decline, increasing fall risk, in cognitively intact individuals aged 60 and older (*N* = 416) during cognitive-load walking tasks, where worse executive attention/processing speed predicts greater stride length reduction [59]. Third, post-HD improvements in speed and stride time suggest acute motor control gains but do not significantly impact conservative gait patterns, such as stride length. This underscores the necessity for sustained interventions targeting a broad spectrum of fall risk factors.

Our study has several limitations. First, the recruitment of older sarcopenic HD patients posed challenges, resulting in a small sample size that may have limited statistical power. As an observational study, we did not perform a power calculation. We were unable to adjust for relevant variables in cases with low events, which could increase the confounding bias in assessing risk estimates. Second, our sample of ESKD patients, enrolled at a single center, was relatively older and had multiple comorbidities (Table 1). The generalizability of our findings may be reduced in younger HD, PD, or KT patients. Third, our cohort was classified based on the questionnaire-based SARC-F without confirming muscle mass through standard body composition analysis, such as bioelectrical impedance or dual X-ray absorptiometry to reduce the assessment burden on vulnerable patients. This may lead to some misclassifications and requires future studies for validation. In addition, the accuracy of body composition measurements in ESKD patients may be compromised by factors such as hydration status and sarcopenic obesity. Fourth, several gait variables, such as gait initiation, turning, or tandem gait under dual-task conditions, were not considered in our study. Further longitudinal work involving repeated gait measurement during maintenance dialysis is warranted. Given the low number of events observed, the mediating role of gait abnormalities in the fall risk of the high-risk sarcopenia group remains unclear. Future enrollment of cases is required for causal mediation analysis to elucidate the association between HD-unique gait signs and fall risk. Finally, a single HD session was used to assess gait, providing a snapshot but not capture the cumulative effects of multiple HD sessions, while minimizing temporal bias. This within-subject design, evaluating gait just four hours apart, controls for day-to-day variability and reduces the influence of extrinsic factors like daily activity or hydration changes, thereby enhancing the reliability of real-time data on HD effects.

## Conclusions

Our study used low-cost wearable accelerometers to automatically extract individual gait features, addressing a gap in the understanding of gait in older HD patients. High-risk sarcopenia, as indicated by a SARC-F score ≥ 4, was associated with various gait abnormalities, including slower gait speed, shorter stride length, prolonged double support phase, and increased time to complete the TUG task. These abnormalities were partially improved by the HD sessions. Importantly, we found that these pathologic gait features were predictive of incident falls, indicating their prognostic value. Our research suggests a paradigm shift from the current practice of treating sarcopenia and secondary prevention after fall to proactive gait analysis in high-risk patients identified by SARC-F. Future large-scale studies are needed to validate the usefulness of spatiotemporal gait assessments for identifying at-risk HD individuals for mobility consequences and developing interventions to improve health outcomes.

## Electronic Supplementary Material

Below is the link to the electronic supplementary material.

Supplementary Material 1

## Data Availability

The data obtained for this study cannot be shared publicly due to its inclusion of confidential information pertaining to research participants. The data used and/or analyzed in the current study are available from the corresponding author on reasonable request.

## Abbreviations

10MWT: 10-meter walk test
BBS: Berg Balance Scale
BMI: body mass index
CC: calf circumference
CI: confidence intervals
CKD: chronic kidney disease
CRP: C-reactive protein
DS: double support
eGFR: estimated glomerular filtration rate
EMR: electronic medical record
ESKD: end-stage kidney disease
EWGSOP: European Working Group on Sarcopenia in Older People
FP: Fried phenotype
GEE: generalized estimating equations
GS: gait speed
HD: hemodialysis
HGS: handgrip strength
ICD: International Classification of Diseases
IMU: inertial measurement unit
IPAQ: International Physical Activity Questionnaire
iPTH: intact parathyroid hormone
KT: kidney transplantation
MCU: microcontroller unit
MET: metabolic equivalent
nPCR: protein catabolic rate
OR: odds ratios
PD: peritoneal dialysis
SARC-F: strength, ambulation, rising from a chair, stair climbing and history of falling
SARC-CalF: SARC-F combined with calf circumference
SBP: systolic blood pressure
SD: standard deviation
SL: stride length
ST: stride time
TUG: Timed-Up and Go
URR: urea reduction rate
US: United States
