# Supplementary material for: Gait abnormalities and longitudinal fall risk in older patients with end-stage kidney disease and sarcopenia
Source: BMC Geriatr. 2024 Nov 13;24:937. doi: 10.1186/s12877-024-05506-z (PMC11559052; doi:10.1186/s12877-024-05506-z)
Supplement: Supplementary file 1 — Supplementary Material 1 [file 12877_2024_5506_MOESM1_ESM.docx]

Supplementary table 1. Analysis of gait parameters in different handgrip strength groups between pre- and post-hemodialysis sessions.

| **Time**  **Gait parameters** | **Low handgrip** ^a^ **strength (N=10)** | | **Normal handgrip strength (N=12)** | |  |
| --- | --- | --- | --- | --- | --- |
|  | **Pre-dialysis** | **Post-dialysis** | **Pre-dialysis** | **Post-dialysis** | **Time x Handgrip strength interaction *p* value** |
| **TUG time (sec)** | 20.3 (17.0-29.4) * | 20.5 (17.3-23.9) † | 11.8 (11.0-17.4) * | 13.3 (11.3-17.3) † | < 0.001 |
| **Gait speed (m/s)** | 0.54 (0.51-0.65) * | 0.60 (0.45-0.69) † | 0.95 (0.70-1.00) * | 0.89 (0.69-0.99) † | 0.079 |
| **Stride time (sec)** | 1.35 (1.09-1.48) | 1.24 (1.04-1.42) | 1.10 (1.06-1.24) | 1.14 (1.05-1.32) | 0.189 |
| **Stride time variability (%)** | 13.2 (8.2-34.9) | 13.1 (11.0-14.5) † | 14.5 (8.8-24.5) | 19.1 (15.4-29.4) † | 0.063 |
| **Stride length (m)** | 0.75 (0.56-0.84) * | 0.69 (0.54-0.83) † | 0.95 (0.78-1.09) * | 1.02 (0.85-1.09) † | 0.854 |
| **Cadence (steps/min)** | 108 (82.8-115.8) | 97.5 (90.3-115.8) | 109.0 (97.0-113.0) | 105.5 (90.8-114.5) | 0.802 |
| **Double support (sec)** | 0.35 (0.32-0.41) | 0.33 (0.29-0.35) | 0.34 (0.31-0.37) | 0.36 (0.31-0.41) | 0.017 |
| **Double support (%)** | 29.8 (27.1-30.1) | 29.6 (23.3-30.0) † | 30.2 (29.7-30.3) | 30.0 (29.8-30.2) † | 0.293 |
| **Stability (%)** | 76.4 (70.9-83.3) * | 78.4 (77.4-86.1) | 62.6 (55.1-72.8) * | 77.3 (60.5-83.6) | 0.159 |
| **Symmetry (%)** | 85.2 (76.8-90.7) * | 88.9 (83.2-93.2) | 64.3 (60.2-79.8) * | 81.6 (77.1-87.1) | 0.057 |

Values for continuous variables given as median (interquartile range).

Abbreviation: TUG, Timed-Up and Go.

**p* < 0.05, comparison between low handgrip strength group and normal handgrip strength group in pre-dialysis.

†*p* < 0.05, comparison between low handgrip strength group and normal handgrip strength group in post-dialysis.

^a^ Low handgrip strength is defined as < 28 kg for men and < 18 kg for women [1].

Supplementary table 2. Analysis of gait parameters in different gait speed groups between pre- and post-hemodialysis sessions

| **Time**  **Gait parameters** | **Low gait speed** ^a^ **(N=18)** | | **Normal gait speed ^a^ (N=4)** | |  |
| --- | --- | --- | --- | --- | --- |
|  | **Pre-dialysis** | **Post-dialysis** | **Pre-dialysis** | **Post-dialysis** | **Time x Gait speed interaction *p* value** |
| **TUG time (sec)** | 18.3 (12.4-23.3) * | 18.8 (12.4-23.1) | 11.0 (9.5-11.4) * | 13.0 (11.3-15.5) | < 0.001 |
| **Stride time (sec)** | 1.21 (1.08-1.45) | 1.24 (1.06-1.38) | 1.03 (0.86-1.14) | 1.14 (0.90-1.29) | 0.244 |
| **Stride time variability (%)** | 13.2 (8.6-26.9) | 14.3 (11.7-20.0) | 14.5 (12.5-46.9) | 19.1 (16.2-42.7) | 0.717 |
| **Stride length (m)** | 0.78 (0.63-0.93) * | 0.78 (0.56-1.04) | 1.05 (0.93-1.15) * | 1.02 (0.87-1.09) | < 0.001 |
| **Cadence (steps/min)** | 107.0 (88.3-113.0) | 97.5 (91.0-113.5) | 115.5 (105.0-137.3) | 105.5 (92.5-134.3) | 0.461 |
| **Double support (sec)** | 0.35 (0.32-0.40) | 0.34 (0.30-0.40) | 0.30 (0.27-0.36) | 0.34 (0.26-0.38) | 0.478 |
| **Double support (%)** | 30.0 (29.6-30.2) | 29.9 (29.5-30.2) | 29.9 (29.6-30.4) | 29.9 (29.7-30.2) | 0.723 |
| **Stability (%)** | 74.7 (61.8-79.9) | 78.5 (72.8-85.8) | 62.6 (59.6-68.5) | 74.0 (63.0-78.0) | 0.542 |
| **Symmetry (%)** | 80.8 (65.2-87.2) | 84.4 (77.5-91.8) | 64.3 (62.1-77.2) | 85.0 (79.1-89.8) | 0.055 |

Values for continuous variables given as median (interquartile range).

Abbreviation: TUG, Timed-Up and Go.

**p* < 0.05, comparison between low gait speed group and normal gait speed group in pre-dialysis.

†*p* < 0.05, comparison between low gait speed group and normal gait speed group in post-dialysis.

^a^ Low gait speed is defined as < 1.00 meter/second as physical performance criteria for sarcopenia diagnosis [1].

Supplementary table 3. Analysis of gait parameters in different calf circumference groups between pre- and post-hemodialysis sessions.

| **Time**  **Gait parameters** | **Low calf** ^a^ **circumference (N=14)** | | **Normal calf circumference (N=8)** | |  |
| --- | --- | --- | --- | --- | --- |
|  | **Pre-dialysis** | **Post-dialysis** | **Pre-dialysis** | **Post-dialysis** | **Time x calf circumference** **interaction *p* value** |
| **TUG time (sec)** | 18.3 (11.4-23.3) | 18.0 (12.4-23.1) | 12.3 (11.3-21.4) | 15.0 (10.9-19.0) | < 0.001 |
| **Gait speed (m/s)** | 0.63 (0.52-0.95) | 0.66 (0.49-0.98) | 0.83 (0.53-0.99) | 0.82 (0.62-0.91) | 0.933 |
| **Stride time (sec)** | 1.14 (1.06-1.40) | 1.08 (0.95-1.35) | 1.13 (1.08-1.40) | 1.29 (1.11-1.35) | 0.148 |
| **Stride time variability (%)** | 14.0 (8.8-31.1) | 15.6 (11.7-26.4) | 14.1 (8.7-23.3) | 15.0 (13.1-18.9) | 0.554 |
| **Stride length (m)** | 0.78 (0.63-0.94) | 0.72 (0.56-1.01) | 0.95 (0.78-1.08) | 1.02 (0.88-1.13) | < 0.001 |
| **Cadence (steps/min)** | 110.0 (91.5-115.8) | 108.0 (93.8-126.3) | 106.0 (86.0-111.0) | 94.5 (89.0-108.3) | 0.481 |
| **Double support (sec)** | 0.34 (0.32-0.39) | 0.32 (0.29-0.35) | 0.34 (0.31-0.41) | 0.37 (0.34-0.41) | 0.473 |
| **Double support (%)** | 30.2 (29.4-30.4) | 29.9 (27.9-30.2) | 29.9 (29.6-30.1) | 29.9 (29.6-30.0) | 0.576 |
| **Stability (%)** | 76.4 (68.5-82.0) * | 78.4 (76.3-85.8) | 63.0 (56.1-67.7) * | 75.4 (54.7-81.5) | 0.284 |
| **Symmetry (%)** | 82.6 (65.7-89.4) | 86.2 (80.5-91.8) | 66.5 (54.2-79.3) | 81.4 (77.2-89.8) | 0.121 |

Values for continuous variables given as median (interquartile range).

Abbreviation: TUG, Timed-Up and Go.

**p* < 0.05, comparison between low calf circumferernce group and normal calf circumferernce group in pre-dialysis.

†*p* < 0.05, comparison between low calf circumferernce group and normal calf circumferernce group in post-dialysis.

^a^ Calf circumference is defined as < 34 cm for men and < 33 cm for women [1].

Supplementary table 4. Analysis of gait parameters in different SARC-CalF scoring groups between pre- and post-hemodialysis sessions.

| **Time**  **Gait parameters** | **Sarcopenia risk (SARC-** **CalF)** | | | |  |
| --- | --- | --- | --- | --- | --- |
|  | **High risk ^a^ (N=11)** | | **Low risk ^a^ (N=11)** | | **Time x SARC-CalF risk interaction *p* value** |
|  | **Pre-dialysis** | **Post-dialysis** | **Pre-dialysis** | **Post-dialysis** |  |
| **TUG time (sec)** | 18.5 (14.0-25.5) | 18.5 (13.5-23.5) | 12.0 (11.0-22.5) | 14.0 (10.5-19.0) | < 0.001 |
| **Gait speed (m/s)** | 0.56 (0.52-0.71) | 0.66 (0.45-0.97) | 0.90 (0.50-1.00) | 0.83 (0.60-0.91) | 0.274 |
| **Stride time (sec)** | 1.12 (1.06-1.46) | 1.06 (0.92-1.26) | 1.16 (1.08-1.32) | 1.30 (1.08-1.36) | 0.035 |
| **Stride time variability (%)** | 20.1 (8.9-34.8) | 14.9 (11.2-27.5) | 13.2 (8.6-19.4) | 15.3 (13.1-19.2) | 0.243 |
| **Stride length (m)** | 0.76 (0.60-0.90) | 0.70 (0.54-0.82) † | 1.00 (0.76-1.10) | 1.04 (0.86-1.10) † | < 0.001 |
| **Cadence (steps/min)** | 113.0 (92.0-124.0) | 113.0 (97.0-130.0) † | 103.0 (90.0-111.0) | 93.0 (88.0-111.0) † | 0.337 |
| **Double support (sec)** | 0.34 (0.32-0.40) | 0.32 (0.28-0.34) | 0.36 (0.31-0.37) | 0.36 (0.32-0.39) | 0.317 |
| **Double support (%)** | 30.2 (29.5-30.5) | 29.8 (29.3-30.2) | 30.0 (29.6-30.2) | 30.0 (29.6-30.2) | 0.462 |
| **Stability (%)** | 76.8 (70.3-82.6) | 78.5 (76.4-84.7) | 64.0 (58.7-73.6) | 77.7 (62.0-82.4) | 0.186 |
| **Symmetry (%)** | 81.5 (67.0-89.1) | 85.0 (81.6-91.4) | 66.5 (62.1-83.8) | 81.6 (77.1-91.4) | 0.159 |

Values for continuous variables given as median (interquartile range).

Abbreviation: TUG, Timed-Up and Go; SARC-CalF, SARC-F combined with calf circumference.

**p* < 0.05, comparison between SARC-CalF ≥ 11 group and SARC-CalF < 11 group in pre-dialysis.

†*p* < 0.05, comparison between SARC-CalF ≥ 11 group and SARC-CalF < 11 group in post-dialysis.

^a^ SARC-CalF classification defines high-risk as a score of ≥ 11 and low-risk as a score of < 11 [1].

Supplementary table 5. Analysis of gait parameters in different balance status categorized by Berg Balance Scale between pre- and post-hemodialysis sessions.

| **Time**  **Gait parameters** | **Poor balance** ^a^ **(N=13)** | | **Better balance (N=9)** | |  |
| --- | --- | --- | --- | --- | --- |
|  | **Pre-dialysis** | **Post-dialysis** | **Pre-dialysis** | **Post-dialysis** | **Time x balance**  **interaction *p* value** |
| **TUG time (sec)** | 21.0 (16.0-27.5) * | 19.0 (16.0-24.3) † | 11.5 (10.3-14.0) * | 12.0 (10.8-16.3) † | < 0.001 |
| **Gait speed (m/s)** | 0.53 (0.49-0.67) * | 0.60 (0.45-0.72) † | 0.98 (0.74-1.01) * | 0.90 (0.80-1.00) † | 0.035 |
| **Stride time (sec)** | 1.32 (1.11-1.48) * | 1.26 (1.06-1.42) | 1.08 (1.02-1.16) * | 1.08 (1.01-1.30) | 0.249 |
| **Stride time variability (%)** | 16.3 (8.6-32.9) | 13.6 (11.6-15.4) | 14.0 (8.80-21.0) | 19.2 (16.8-26.4) | 0.339 |
| **Stride length (m)** | 0.76 (0.54-0.86) * | 0.70 (0.54-0.90) † | 1.00 (0.86-1.10) * | 1.04 (0.91-1.10) † | 0.705 |
| **Cadence (steps/min)** | 107.0 (82.5-113.0) | 97.0 (88.5-113.0) | 111.0 (103.0-116.5) | 111 (91.5-119.0) | 0.662 |
| **Double support (sec)** | 0.36 (0.33-0.42) | 0.33 (0.29-0.40) | 0.32 (0.30-0.37) | 0.35 (0.31-0.39) | 0.009 |
| **Double support (%)** | 29.9 (29.3-30.3) | 29.8 (26.6-30.0) † | 30.2 (29.6-30.3) | 30.2 (29.7-30.2) † | 0.177 |
| **Stability (%)** | 75.8 (65.9-80.9) | 77.7 (57.1-81.6) | 63.1 (57.0-74.0) | 78.7 (73.9-86.5) | < 0.001 |
| **Symmetry (%)** | 81.5 (66.7-89.8) | 85.0 (74.4-92.2) | 66.5 (61.7-82.3) | 85.0 (77.4-89.6) | 0.005 |

Values for continuous variables given as median (interquartile range).

Abbreviation: TUG, Timed-Up and Go.

**p* < 0.05, comparison between poor balance group and better balace group in pre-dialysis.

†*p* < 0.05, comparison between poor balance group and better balace group in post-dialysis.

^a^ Balance state is categorized as poor for scores below 45 points on the Berg Balance Scale and as better for scores ≥ 45 points [2].

Supplementary table 6. Analysis of gait parameters in different frail state groups between pre- and post-hemodialysis sessions.

| **Time**  **Gait parameters** | **Frail ^a^ (N=11)** | | **Prefrail ^a^ (N=8)** | | **Robust ^a^ (N=3)** | |  |
| --- | --- | --- | --- | --- | --- | --- | --- |
|  | **Pre-dialysis** | **Post-dialysis** | **Pre-dialysis** | **Post-dialysis** | **Pre-dialysis ^b^** | **Post-dialysis ^b^** | **Time x frail**  **interaction *p* value** |
| **TUG time (sec)** | 22.5 (18.0-29.5) * | 22.0 (18.5-25.0) † | 12.3 (11.1-17.4) * | 15.0 (12.0-17.3) † | 11.0 (9.5-11.0) * | 11.0 (8.5-12.5) † | < 0.001 |
| **Gait speed (m/s)** | 0.52 (0.48-0.63) * | 0.60 (0.44-0.66) † | 0.86 (0.70-1.00) * | 0.86 (0.69-0.91) † | 0.98 (0.94-1.08) * | 1.00 (0.97-1.04) † | 0.077 |
| **Stride time (sec)** | 1.32 (1.10-1.46) | 1.26 (1.08-1.42) | 1.12 (1.07-1.27) | 1.24 (1.07-1.35) | 1.06 (0.82-1.12) | 1.04 (0.84-1.06) | 0.663 |
| **Stride time variability (%)** | 16.3 (8.7-34.8) | 13.6 (11.9-23.2) | 10.5 (8.2-23.4) | 18.7 (9.3-19.5) | 15.8 (14.0-27.7) | 32.5 (15.6-50.5) | 0.478 |
| **Stride length (m)** | 0.74 (0.48-0.80) * | 0.68 (0.54-0.86) † | 0.95 (0.82-1.10) * | 1.02 (0.87-1.09) † | 1.04 (0.9-1.04) * | 1.04 (0.82-1.10) † | 0.058 |
| **Cadence (steps/min)** | 95.0 (82.0-113.0) | 96.0 (85.0-111.0) | 111.0 (103.0-118.3) | 101.5 (90.8-112.5) | 113.0 (107.0-143.0) | 115.0 (113.0-142.0) | 0.681 |
| **Double support (sec)** | 0.37 (0.32-0.42) | 0.34 (0.29-0.41) | 0.34 (0.31-0.37) | 0.36 (0.33-0.39) | 0.31 (0.27-0.33) | 0.30 (0.24-0.31) | 0.011 |
| **Double support (%)** | 29.9 (29.5-30.2) | 29.8 (29.3-30.0) | 29.9 (29.6-30.2) | 30.1 (29.6-30.3) | 30.4 (30.2-30.5) | 29.8 (29.8-30.2) | 0.228 |
| **Stability (%)** | 76.0 (67.8-82.6) | 78.3 (62.0-84.7) | 62.6 (56.1-70.7) | 78.7 (76.4-83.6) | 70.3 (38.9-81.8) | 60.0 (25.6-89.1) | < 0.001 |
| **Symmetry (%)** | 81.5 (67.0-90.5) | 85.0 (81.2-93.1) | 64.3 (61.5-82.0) | 83.3 (77.2-90.4) | 80.8 (59.8-83.8) | 85.0 (67.5-87.8) | 0.006 |

Values for continuous variables given as median (interquartile range).

Abbreviation: TUG, Timed-Up and Go.

*p < 0.05, comparison among frail group, prefrail group and robust group in pre-dialysis.

†p < 0.05, comparison among frail group, prefrail group and robust group in post-dialysis.

^a^ Frailty is classified according to the Fried phenotype as frail (score ≥ 3), pre-frail (score 1-2), and robust (score 0) [3].

^b^ Because there were only 3 data in the group, the values for continuous variables given as median (minimum – maximum).

Supplementary table 7. Primary and secondary outcomes of the older hemodialysis patients and comparisons between different risk of sarcopenia groups.

| **Event, N (%)** | **All (N=22)** | **Sarcopenia risk (SARC-F)** | | ***p* value** |
| --- | --- | --- | --- | --- |
|  |  | **High risk ^a^ (N=9)** | **Low risk ^a^ (N=13)** |  |
| Outcome - no. (%) |  |  |  |  |
| Any injurious falls | 10 (45.5) | 7 (77.8) | 3 (23.1) | 0.027 |
| Incident fall ^b^ | 7 (31.8) | 4 (44.4) | 3 (23.1) | 0.376 |
| Recurrent fall ^c^ | 3 (13.6) | 3 (33.3) | 0 (0.0) | 0.055 |
| Single fall ^d^ | 6 (27.3) | 4 (44.4) | 2 (15.4) | 0.178 |
| Multiple falls ^e^ | 4 (18.2) | 3 (33.3) | 1 (7.7) | 0.264 |

Abbreviations and definitions: SARC-F, strength, ambulation, rising from a chair, stair climbing and history of falling.

^a^ Sarcopenia risk is categorized as high-risk SARC-F ≥ 4 and low-risk SARC-F < 4 [1].

^b^ Incident fall: first fall without previous history.

^c^ Recurrent Fall: fall occurrence with prior history.

^d^ Single Fall: one fall event during follow-up.

^e^ Multiple Falls: more than one fall events during follow-up.

Supplementary table 8. Prediction accuracy of longitudinal falls for SARC-F and other individual risk profile.

|  | Univariable OR | *p* value | Multivariable OR^*^ | *p* value | Sensitivity (%) | Specificity (%) | Youden index | PPV (%) | NPV (%) |
| --- | --- | --- | --- | --- | --- | --- | --- | --- | --- |
| High risk by SARC-F ^a^ | 11.67 (1.53-89.12) | 0.018 | 7.36 (0.80-68.09) | 0.079 | 70.0 | 83.3 | 0.533 | 77.8 | 76.9 |
| Low handgrip strength ^b^ | 7.00 (1.07-45.90) | 0.043 | 10.42 (0.94-115.22) | 0.056 | 70.0 | 75.0 | 0.450 | 70.0 | 75.0 |
| Low calf circumference ^c^ | 1.67 (0.28-9.82) | 0.572 | 1.04 (0.11-9.98) | 0.976 | 70.0 | 41.7 | 0.117 | 50.0 | 62.5 |
| High risk by SARC-CalF ^d^ | 2.10 (0.38-11.59) | 0.395 | 2.03 (0.22-18.58) | 0.530 | 60.0 | 58.3 | 0.183 | 54.5 | 63.6 |
| Poor balance ^e^ | 18.00 (1.65-196.31) | 0.018 | 25.53 (1.46-446.69) | 0.026 | 90.0 | 66.7 | 0.567 | 69.2 | 88.9 |

Gait speed and frailty were not included in fall risk prediction due to the limited number of robust (N=3) and normal gait speed (N=4) reference participants.

Abbreviations: SARC-F, strength, ambulation, rising from a chair, stair climbing and history of falling; SARC-CalF, SARC-F combined with calf circumference; OR, odds ratio; PPV, postive predictive values; NPV, negative predictive values.

^*^ Adjusted for age, sex

^a^ SARC-F classification is defined as high-risk with a score ≥ 4 and low-risk is defined as a score < 4 [1].

^b^ Low handgrip strength is defined as < 28 kg for men and < 18 kg for women [1].

^c^ Calf circumference is defined as < 34 cm for men and < 33 cm for women [1].

^d^ SARC-CalF classification defines high risk as a score of ≥ 11 and low risk as a score of < 11 [1].

^e^ Balance state is categorized as poor for scores below 45 points on the Berg Balance Scale and as better for scores ≥ 45 [2].

| Author | Year | Country | Design | ESKD Modality | Number | Age (years) | Relation to HD session | Equipment, methodology | Attachment of sensors |
| --- | --- | --- | --- | --- | --- | --- | --- | --- | --- |
| Lockhart  [4] | 2010 | USA | Cross sectional | HD | 5 | NA | Pre- and Post-HD | Portable, non-invasive TEMPO: wireless body sensor network platform | 5 TEMPO nodes (one on each ankle, one on each wrist, and one on the sacrum) |
| Soangra  [5] | 2013 | USA | Cross sectional | HD | 6 | 54 ± 4 | Pre- and Post-HD | Portable, non-invasive TEMPO: wireless body sensor network platform. | 5 IMU units (one on each lateral side of shank, and one on the sacrum,  one on each wrist) |
| Zemp  [6] | 2021 | Switzerland | Longitudinal (2-year duration) | HD | 14 | 72.4 ± 5.4 | NA | Triaxial accelerometer (DynaPort MiniMod,  McRoberts, The Hague, NL) | Affixed to the lower trunk, between the left/right spina iliaca posterior superior by elastic belt |
| Zemp  [7] | 2022 | Switzerland | Cross sectional | HD | 9 | 75.9 ± 7.2 | NA | Triaxial accelerometer (DynaPort MiniMod,  McRoberts, The Hague, NL) | Affixed by an elastic belt to the lower trunk, between the left/right spina iliaca posterior superior |
| Zanotto  [8] | 2023 | UK | Cross sectional | HD | 59 | 62.3 ± 14.9 | NA | ActivPAL uniaxial accelerometer (PAL Technologies Ltd, Glasgow, UK) | Wear on the anterior aspect of the thigh |

Supplementary table 9. Characteristics of studies using triaxial accelerometers for quantitative gait assessment in ESKD patients.

Supplementary Table 9. Characteristics of studies using triaxial accelerometers for quantitative gait assessment in ESKD patients (continued).

| Author | Task | Quantitative gait parameters | Setting | Clinical factors | Outcome |
| --- | --- | --- | --- | --- | --- |
| Lockhart  [4] | Posturo-Locomotion-Manual test, TUG | Local dynamic stability calculated from original time series data (AP acceleration) | NA | Impact of HD session | Similar local dynamic stability in pre- and post-HD sessions |
| Soangra  [5] | Sit to walk | Peak AP acceleration, peak flexion/extension angular velocity, peak flexion/extension acceleration, initial flexion angular acceleration, time to generate peak flexion/extension angular velocity, time to STW completion | 3-meter away from the chair | Impact of HD session | HD therapy decreased STW parameters (flexion acceleration prior to seat-off), increased event timing sequence |
| Zemp  [6] | Walk while counting down from 100 in steps of 3 trials | Gait speed, cadence, step, step/stride time, step/stride length, Coefficient of variation | 14 meters pathway | HD vintage | Pathological performance of gait movement variability did not alter in trials after entering HD. |
| Zemp  [7] | Walk while counting down from 100 in steps of 3 trials | Gait speed, cadence, step, step/stride time, step/stride length, Coefficient of variation | 14 meters pathway | Renal function | Reduction in gait performance (gait speed and stride length, increased gait variability) correlated with CKD severity. |
| Zanotto  [8] | Daily walking hours | number of daily steps, number of daily sit- to-stand transfers, cadences | Daily walking | Frail | Frail participants performed a lower number of daily steps and sit-to-stand transitions. |

Abbreviations: USA, United States of America; UK, United Kingdom; ESKD, end stage kidney disease; HD, hemodialysis; TUG, Timed-Up and GO; AP, anterio-posterior; IMU, inertial measurement units; STW, sit to walk; CKD, chronic kidney injury; TEMPO, Technology-Enabled Medical Precision Observation; NA, not available.

**Reference**

1. Chen LK, Woo J, Assantachai P, Auyeung TW, Chou MY, Iijima K, Jang HC, Kang L, Kim M, Kim S *et al*: **Asian Working Group for Sarcopenia: 2019 Consensus Update on Sarcopenia Diagnosis and Treatment**. *J Am Med Dir Assoc* 2020, **21**(3):300-307 e302.

2. Lima CA, Ricci NA, Nogueira EC, Perracini MR: **The Berg Balance Scale as a clinical screening tool to predict fall risk in older adults: a systematic review**. *Physiotherapy* 2018, **104**(4):383-394.

3. Fried LP, Tangen CM, Walston J, Newman AB, Hirsch C, Gottdiener J, Seeman T, Tracy R, Kop WJ, Burke G *et al*: **Frailty in older adults: evidence for a phenotype**. *J Gerontol A Biol Sci Med Sci* 2001, **56**(3):M146-156.

4. Lockhart TE, Barth AT, Zhang X, Songra R, Abdel-Rahman E, Lach J: **Portable, Non-Invasive Fall Risk Assessment in End Stage Renal Disease Patients on Hemodialysis**. *ACM Trans Comput Hum Interact* 2010:84-93.

5. Soangra R, Lockhart TE, Lach J, Abdel-Rahman EM: **Effects of hemodialysis therapy on sit-to-walk characteristics in end stage renal disease patients**. *Ann Biomed Eng* 2013, **41**(4):795-805.

6. Zemp DD, Giannini O, Quadri P, Rabuffetti M, Tettamanti M, de Bruin ED: **Signatures of Gait Movement Variability in CKD Patients Scheduled for Hemodialysis Indicate Pathological Performance Before and After Hemodialysis: A Prospective, Observational Study**. *Front Med (Lausanne)* 2021, **8**:702029.

7. Zemp DD, Giannini O, Quadri P, Rabuffetti M, Tettamanti M, de Bruin ED: **Gait disorders in CKD patients: muscle wasting or cognitive impairment? A cross-sectional pilot study to investigate gait signatures in Stage 1-5 CKD patients**. *BMC Nephrol* 2022, **23**(1):72.

8. Zanotto T, Mercer TH, van der Linden ML, Traynor JP, Koufaki P: **Use of a wearable accelerometer to evaluate physical frailty in people receiving haemodialysis**. *BMC Nephrol* 2023, **24**(1):82.
